# A concise review on the role of MIR100HG in human disorders

**DOI:** 10.1111/jcmm.17875

**Published:** 2023-07-24

**Authors:** Soudeh Ghafouri‐Fard, Atefeh Harsij, Hossein Farahzadi, Bashdar Mahmud Hussen, Mohammad Taheri, Majid Mokhtari

**Affiliations:** ^1^ Department of Medical Genetics, School of Medicine Shahid Beheshti University of Medical Sciences Tehran Iran; ^2^ Phytochemistry Research Centre Shahid Beheshti University of Medical Sciences Tehran Iran; ^3^ Department of Clinical Analysis, College of Pharmacy Hawler Medical University Erbil Iraq; ^4^ Urology and Nephrology Research Centre Shahid Beheshti University of Medical Sciences Tehran Iran; ^5^ Institute of Human Genetics Jena University Hospital Jena Germany; ^6^ Skull Base Research Centre, Loghman Hakim Hospital Shahid Beheshti University of Medical Sciences Tehran Iran

**Keywords:** cancer, cardiomyopathy, fibrosis, lncRNA, MIR100HG

## Abstract

MIR100HG is a long non‐coding RNA (lncRNA) encoded by a locus on chr11:122,028,203‐122,556,721. This gene can regulate cell proliferation, apoptosis, cell cycle transition and cell differentiation. MIR100HG was firstly identified through a transcriptome analysis and found to regulate differentiation of human neural stem cells. It is functionally related with a number of signalling pathways such as TGF‐β, Wnt, Hippo and ERK/MAPK signalling pathways. Dysregulation of MIR100HG has been detected in a diversity of cancers in association with clinical outcomes. Moreover, it has a role in the pathophysiology of dilated cardiomyopathy, intervertebral disk degeneration and pulmonary fibrosis. The current study summarizes the role of these lncRNAs in human disorders.

## 1 INTRODUCTION

Long noncoding RNAs (lncRNAs) are transcripts with sizes more than 200 nucleotides that do not encode proteins. These transcripts play critical regulatory role in numerous biological events through various mechanisms, including regulation of chromatin structure, interaction with other RNA molecules, sequestering miRNAs and serving as scaffolds for establishment of protein complexes.^1^ Genes encoding lncRNAs can be categorized according to their relation with other genes in the genome. MicroRNA‐host‐gene‐derived lncRNAs (MIRHGs) are a group of lncRNAs derived from miRNA host genes as a result of pre‐miRNA processing.^2^ About 17% of miRNAs are estimated to be produced by MIRHGs.^3^ The processing of MIRHGs is functionally related with synthesis of the encoded miRNAs. Notably, MIRHGs have been shown to play important functions in fundamental cellular events and pathogenesis of diseases.^2^

The molecular crosstalk between lncRNAs and miRNAs plays a fundamental role in the development of a number of diseases mainly through lncRNA/miRNA sponging mechanisms. The sponging effect of lncRNAs on miRNAs attenuates the repression of mRNAs by miRNAs.^4^ These effects have been best studied in cancer where lncRNA‐miRNA axes regulate apoptosis and autophagy. Moreover, these axes influence tumour metastases through regulation of epithelial‐mesenchymal transition (EMT) and expression of matrix metalloproteinase.^5^

**FIGURE 1:**
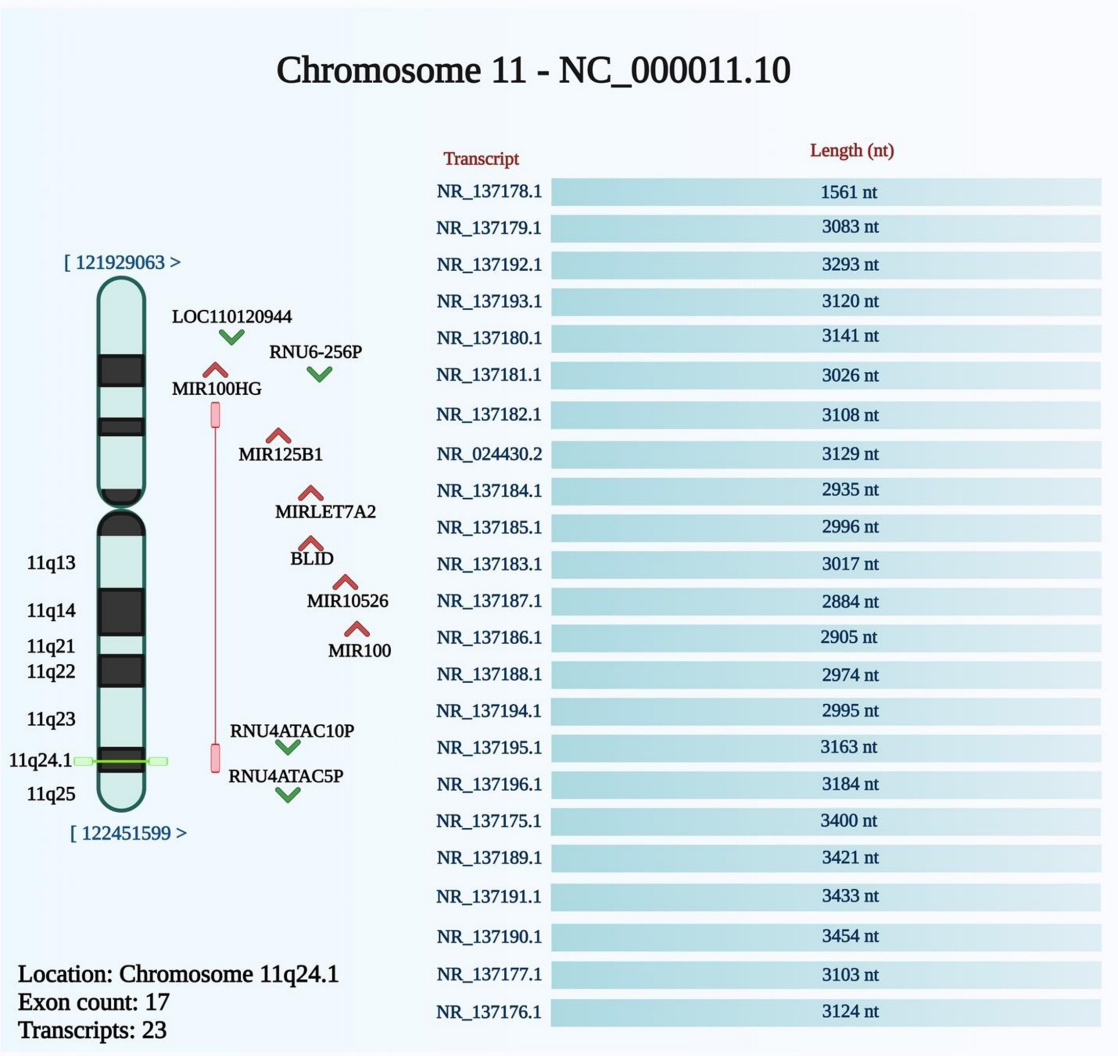
The schematic diagram represented the chromosomal location of the MIR100HG, which is encoded by a gene located on chromosome 11q24.1. From the NCBI database (http://www.ncbi.nlm.nih.gov/gene/399959), the genomic context of the MIR100HG was extracted.

MIR100HG, alternatively named as Mir‐100‐Let‐7a‐2‐Mir‐125b‐1 Cluster Host Gene, is encoded by a gene located on chr11:122,028,203‐122,556,721 (Figure 1). The gene encoding this lncRNA has 17 exons. The intronic coding region in this lncRNA has a role as a pro‐apoptotic element. This role is exerted through influencing the caspase‐dependent mitochondrial apoptotic axis.^6^ Alternative splicing events can give rise to more than 100 transcripts for MIR100HG with sizes ranging from 242 to 7061 bp.^7^ The main locus of MIR100HG is the nucleus with also being detected in the cytoplasm.^8^

This gene produces lncRNAs that regulate cell proliferation. MIR100HG was firstly identified through a transcriptome analysis^9^ and found to be a crucial regulator of differentiation of human neural stem cells.^10^ Several transcript variants are produced from this gene through alternative promoter usage and splicing. Notably, some of these variants have a role in promotion of cell growth, while others are negative regulators of cell division.

A number of spliced and stable lncRNAs that are produced by MIR100HG exhibit up‐regulation during the G1 phase of the cell cycle. It has been shown that depletion of these lncRNAs in human cells leads to abnormalities in the progression of cell cycle without changing the expression amounts of MIR100HG‐encoded miRNAs. The absence of significant alterations in the expression of mature MIR100HG‐encoded miRNAs during the cell cycle might be explained by their enhanced stability. However, the nuclear‐restricted MIR100HG has a dynamic expression pattern and differential stability during the cell cycle. These findings indicate MIR100HG‐encoded lncRNAs might contribute in cell cycle progression.^11^ Functional studies have shown interaction between MIR100HG and HuR/ELAVL1 as well as numerous HuR‐target transcripts.^11^ The current study summarizes the role of these lncRNAs in human disorders.

## 2 MALIGNANT DISORDERS

### 2.1 Cell line studies

MIR100HG has been shown to be over‐expressed in acute megakaryoblastic leukaemia (AMKL) blasts. MIR100HG transcripts are principally localized in the nucleus parallel with miRNA cluster. MIR100HG silencing has obstructed leukaemic growth of AMKL cell lines. This study has indicated the role of MIR100HG in the haematopoiesis and its oncogenic effect in the evolution of myeloid leukaemia.^12^ In order to evaluate function of MIR100HG in AMKL, this lncRNA has been down‐regulated in a human cell line derived from this type of malignancy (M‐07e) using antisense LNA GapmeRs. This study have indicated that suppression of MIR100HG can inhibit proliferation of M‐07e cells, induce apoptosis and necrosis and enhance expression of TGF‐β.^13^

**FIGURE 2:**
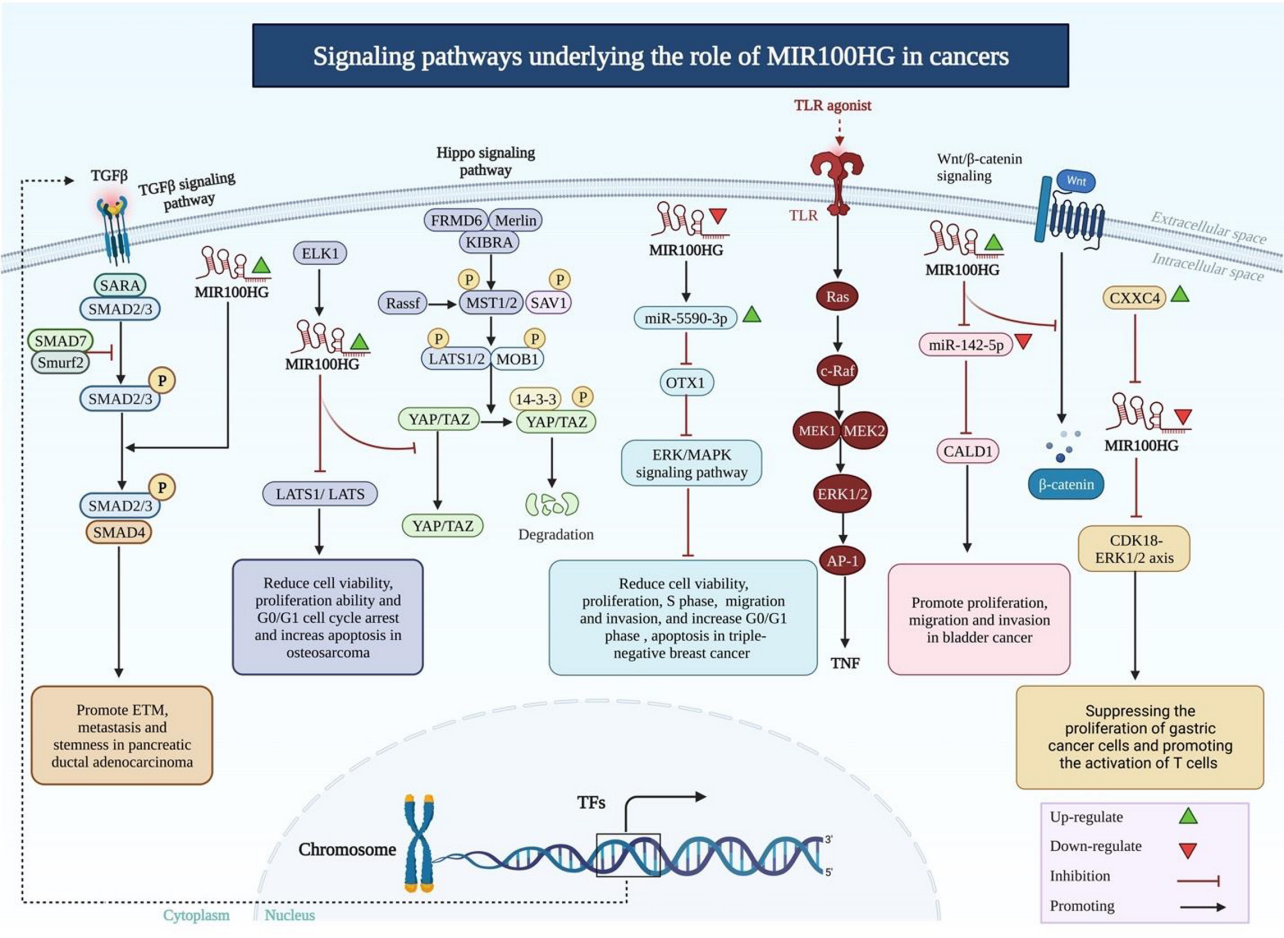
Schematic representation of the key functions of MIR100HG lncRNA. It plays a major role in a number of malignancies by interfering with critical intracellular signalling pathways. TGF‐β, Hippo and Wnt/β‐catenin are among signalling pathways that are regulated by this lncRNA. miR‐142‐5p/CALD1 and miR‐5590‐3p/OTX1 are among miRNA/mRNA axes that are regulated by MIR100HG.

An experiment in bladder cancer cells has shown that up‐regulation of MIR100HG efficiently promotes proliferation, migration and invasion of the 5637 bladder carcinoma cells, inhibits expression of miR‐142‐5p and induces expression of CALD1. Mechanistically, miR‐142‐5p can inhibit expression of CALD1 in these cells via a direct interaction and reverses the effects of MIR100HG over‐expression in proliferation of bladder cancer cells (Figure 2). Taken together, MIR100HG has a regulatory role on expression of CALD1 through targeting miR‐142‐5p.^14^

Constitutive up‐regulation of β‐catenin in colorectal cancer cells has been found to decrease levels of primary and mature MIR100HG transcripts, while obstruction of β‐catenin activity has led to enhancement of their expression. Further studies have confirmed the ability of β‐catenin/TCF4 in binding with MIR100HG promoter. Besides, forced over‐expression of β‐catenin has led to lessening of the enrichment of H3K27Ac on this promoter. Besides, HDAC6 recruitment on the MIR100HG promoter has led to reduction of H3K27Ac enrichment through a β‐catenin‐dependent route. Up‐regulation of MIR100HG has led to G0‐G1 arrest and suppression of cell proliferation through increasing p57 levels. Cumulatively, ectopic β‐catenin can repress transcription of MIR100HG via HDAC6‐mediated histone modifications.^15^ Another study in colorectal cancer cells has shown that the interplay between MIR100HG and hnRNPA2B1 can increase m (6)A‐dependent stability of TCF7L2 transcripts and facilitate progression of colorectal cancer.^16^

In the context of gastric cancer, CXXC finger protein 4 has been shown to inhibit the CDK18‐ERK1/2 axis to inhibit the immune escape through modulation of ELK1/MIR100HG pathway.^17^

MIR100HG silencing has been shown to attenuate progression of hepatocellular carcinoma through modulation of miR‐146b‐5p/CBX6 axis.^18^ In laryngeal squamous cell carcinoma, MIR100HG exerts its oncogenic effects via down‐regulating expression of miR‐204‐5p.^19^ In osteosarcoma cells, expression of MIR100HG has been shown to be induced by ELK1. This lncRNA enhances progression of osteosarcoma through decreasing expressions of LATS1 and LATS2 via epigenetical mechanisms.^20^

MIR100HG expression has been found to be induced by TGFβ in different cancer types. MIR100HG silencing has decreased activity of TGFβ signalling and reduced expression of TGFβ‐target genes. Besides, MIR100HG silencing has suppressed motility of both normal and cancer cells and increased the cytotoxic effects of cytostatic drugs.^21^

Table 1 shows the impact of MIR100HG in the cancer cell lines.

**TABLE 1:** Function of MIR100HG in different types of tumour cell lines.

Tumour type	Cell line	Expression	Targets/Regulators and signalling pathways	Function	Reference
Acute megakaryoblastic leukaemia (AMKL)	TCLs: CMK, Meg‐01, K562, HT1080, 293 T	High (AMKL cell lines vs. other leukaemic cell lines)	_	↓MIR100HG: ↓Proliferation, ↓colony formation ability, ↑subG1 phase, ↓s phase, ↑Annexin+ apoptotic cells, ↑CD36+ cells and ↓CD41+ cells (changing surface markers expression)	^12^
	TCL: M‐07e	_	TGF‐β	↓MIR100HG: ↓cell viability, ↑apoptosis, ↑necrosis	^13^
Bladder cancer	Human bladder cancer cells	_	miR‐142‐5p/CALD1	↑MIR100HG: ↑proliferation, ↑migration, ↑invasion	^14^
	TCLs: RT‐4, UM‐UC‐3, 5637, SCaBER, SW780, T24, MGH‐U3; NCL: SV‐HUC‐1	Low (TCLs vs. NCL)	_	_	^22^
Colorectal cancer	TCLs: RKO, SW480, SW620, HT‐29, HCT‐8, CCD841, NCM460, DLD‐1, HEK‐293, DiFi	_	β‐catenin/TCF4/HDAC6 (regulators: inhibit MIR100HG transcription) p57 (target of MIR100HG)	↑MIR100HG: ↓Cell growth, ↓proliferation, ↑G0/G1 cell cycle arrest	^15^
	TCLs: LoVo, DLD‐1, RKO, SW48, HCT8, HCT116; NCL: FHC	High (TCLs vs. NCL) Highest: HCT116, lowest: LoVo)	_	↓MIR100HG (in HCT116): ↓migration, ↓invasion ↑MIR100HG (in LoVo): ↑migration, ↑invasion	^23^
	TCLs:HCA‐7 (CTX‐sensitive CC cells and CTX‐resistant CC‐CR cells), NCI‐H508, Caco‐2, SW403, SW948, HT29, SK‐CO‐1, DLD‐1, SW480, SW837	High (CC‐CR cells vs. CC cells)	hnRNPA2B1/ TCF7L2, Wnt signalling pathway	↑MIR100HG: ↓E‐cadherin, ↑mesenchymal genes, ↑EMT‐TFs (↑EMT), ↑CTX‐resistance, ↑migration, ↑invasion	^16^
Gastric cancer	TCLs: MGC‐803, SGC7901, BGC‐823, AGS NCL: GES‐1	High (TCLs vs. NCL)	_	↓MIR100HG: ↓cell proliferation, ↓migration, ↓invasion	^24^
	TCLs: SGC‐7901, BGC‐823	_	_	↓MIR100HG: ↓cell mitosis (proliferation), ↓migration, ↓invasion	^25^
	TCLs: SGC‐7901, BGC‐823, SNU‐1, HGC‐27; NCL: GES‐1	High (TCLs vs. NCL)	CXX4/ELK1(MIR100HG regulator) Targets: CDK18‐ERK1/2	↑CXX4 → ∆MIR100HG: ↓ CDK18‐ERK1/2 → ↓proliferation, ↑ T cells activation	^17^
Hepatocellular carcinoma	TCLs: Hep3B, HepG2, SK‐HEP1, Huh7 NCL: LO2	High (TCLs vs. NCL)	miR‐146b‐5p/CBX6	↓MIR100HG: ↓cell viability, ↓migration, ↓invasion	^18^
Laryngeal squamous cell carcinoma	TCL: UM‐SCC‐17A	_	miR‐204‐5p	↑MIR100HG: ↑proliferation, ↑migration, ↑invasion	^19^
Osteosarcoma	TCLs: MG‐63, MNNG/HOS, U2OS, 143B; NCL: Hfob1.19	High (TCLs vs. NCL)	ELK1 EZH2/ LATS1/ S2 Hippo signalling pathway (inactivation)	↓MIR100HG: ↓cell viability, ↓proliferation ability, ↑G0/G1 cell cycle arrest, ↑apoptosis	^20^
Pancreatic ductal adenocarcinoma	BxPC‐3, PANC‐1, COLO357, S2‐007, S2‐028, CHX45	_	TGF‐β, SMAD2/3	TGF‐β → SMAD2/3 → ↑MIR100HG → ↑miR‐100 and miR‐125b: ↑ETM, ↑metastasis, ↑stemness*	26, 27
Triple‐negative breast cancer (TNBC)	TCLs: MDA‐MB‐231, MDA‐MB‐453, MDA‐MB‐468, MDA‐MB‐415; NCLs: HEK‐293 T, MCF‐10A	High (TCLs vs. NCLs)	miR‐5590‐3p/OTX1, ERK/MAPK signalling pathway	↓MIR100HG: ↓cell viability, ↓proliferation, ↑G0/G1 phase, ↓S phase, ↑apoptosis, ↓migration, ↓invasion	^28^
	TNBC cell lines: MDA‐MB‐231, BT549, HCC1806, HCC1937 Other breast cancer cell lines: MCF7, T47D, SKBR3	High (TNBC cell lines vs. other cell lines	CDKN1B (gene locus of p27)	↑MIR100HG: ↑Cell growth, ↑s phase, ↓p27 and p21, ↑cyclin D1 ↓MIR100HG: ↓Cell proliferation, ↑G1 phase arrest, ↑p27 and p21, ↓cyclin D1	^29^

Abbreviations: CTX: cetuximab; CC‐CR: CTX‐resistant colorectal cancer cell; EMT: epithelial‐mesenchymal transition; NCL: normal cell line; TCLs: tumour cell lines; TFs: transcription factors.

* The study focused on miR‐125b and miR‐100 instead of MIR100HG.

### 2.2 Animal studies

While a single animal study has shown that up‐regulation of MIR100HG reduces growth of colorectal cancer cells in nude mice,^15^ another in vivo animal study has revealed that up‐regulation of MIR100HG can promote migration and invasion of colorectal carcinoma and development of liver metastasis in mice.^23^ Consistent with the latter study, MIR100HG silencing in colorectal cancer cells has reduced lung metastases and enhanced survival of animals in another in vivo study.^16^ Two other in vivo studies in animal models of triple negative breast cancer have confirmed the oncogenic roles of MIR100HG and revealed possible targets for this lncRNA. In vivo experiments using stably silenced MIR100HG MDA‐MB‐231 cells have shown attenuation of tumour growth and reduction in tumour weight. Moreover, these experiments have indicated down‐regulation of OTX1 and up‐regulation of miR‐5590‐3p expression after MIR100HG silencing.^28^ Moreover, another xenograft assay using MDA‐MB‐231 cells has confirmed the effects of MIR100HG silencing on impairment of tumour growth^29^ (Table 2).

**TABLE 2:** Effects of MIR100HG in cancer animal models.

Tumour type	Animal models	Results	Reference
Colorectal cancer	Male BALB/c nude mice	↑MIR100HG: ↓Tumour growth, ↓tumour volume, ↑p57	^15^
	Nude mice	↑MIR100HG: ↑liver metastatic colonies ↓MIR100HG:: ↓liver metastatic colonies	^23^
	Athymic nude mice	↓MIR100HG: ↓lung metastasis, ↑survival	^16^
Triple‐negative breast cancer	Male BALB/c athymic nude mice	↓MIR100HG: ↓tumour volume, ↓tumour weight, ↓OTX1 protein, ↑miR‐5590‐3p, ↓phosphorylated MAPK, MEK, ERK, JNK proteins	^28^
	Female nude mice	↓MIR100HG ↓tumour growth, ↑p27 protein level	^29^

### 2.3 Experiments in clinical samples

MIR100HG has been among differentially expressed lncRNAs in bladder cancer versus non‐cancerous samples as identified by a high throughput sequencing strategy. Notably, survival prognosis analyses, Cox analysis and additional in silico methods have shown the importance of MIR100HG/miR‐142‐5p/CALD1 axis in bladder cancer development. Expression studies in clinical samples have shown up‐regulation of MIR100HG and CALD1 and down‐regulation of miR‐142‐5p. Besides, expression of MIR100HG has been positively correlated with grade of tumours and clinical outcome of this type of cancer.^14^ MIR100HG has also been among immune‐related lncRNAs whose expressions have been correlated with prognosis of patients with bladder cancer.^22^

Assessment of TCGA expression data and clinical samples from a different cohort of patients with colorectal cancer has revealed negative correlation between MIR100HG levels and those of target genes from β‐catenin pathway. In addition, HDAC6 levels have been increased and inversely correlated with levels of MIR100HG in clinical samples of colorectal carcinoma.^15^ MIR100HG expression has also been found to be enhanced in colorectal cancer compared with corresponding normal samples in another patient's cohort. Particularly, MIR100HG over‐expression has been more prominent in advanced colorectal cancer compared with in early stage samples. Based on the Kaplan–Meier analyses, patients with this type of cancer and high levels of MIR100HG expression have poorer disease‐free and overall survival times compared with those having low levels of MIR100HG.^23^ Contrary to these findings, an analysis in GEPIA database has shown down‐regulation of MIR100HG in adenocarcinomas of colon and rectum compared with normal tissues.^30^

MIR100HG expression has also been reported to be elevated in gastric cancer tissues compared with normal gastric mucosa tissues. More importantly, over‐expression of MIR100HG has been associated with clinical stage, tumour invasion and both lymph and distant metastases in these patients. Besides, MIR100HG levels have been negatively correlated with clinical outcome in TCGA gastric cancer samples and another cohort of patients.^24^ An in‐depth study of publicly available data of gastric cancer samples has led to identification of 83 differently expressed lncRNAs in gastric cancer and construction of co‐expression networks between lncRNAs and mRNAs. Notably, MBNL1‐AS1, HAND2‐AS1 and MIR100HG have been at the core of this network. Further analyses have suggested MIR100HG as a possible prognostic factor for gastric cancer. Moreover, patients having higher levels of MIR100HG have exhibited poorer prognosis.^25^

An experiment in laryngeal squamous cell carcinoma samples has shown elevation of MIR100HG and under‐expression of miR‐204‐5p. Moreover, expression of MIR100HG has been correlated with AJCC stage. Notably, expression levels of MIR100HG and miR‐204‐5p have been correlated with each other in tumour tissues but not in adjacent non‐cancerous tissues.^19^ Table 3 reviews the role of MIR100HG in carcinogenesis based on the results of expression assays in clinical samples.

**TABLE 3:** Expression and function of MIR100HG in clinical samples from cancer patients.

Tumour type	Samples	Expression(tumour vs. normal)	Tumour‐associated polymorphism in MIR100HG	Kaplan–Meier analysis (impact of MIR100HG dysregulation)	Univariate/multivariate cox regression analysis	Association of MIR100HG dysregulation with clinicopathologic characteristics	Reference
Bladder cancer	20 PTAs	Upregulated	_	_	_	Tumour histological grade (T stage and clinical stage)	^14^
	GEO database	_	_	Poorer survival	_	T stage	^31^
	49 PTAs +TCGA data	Downregulated	_	_	_	_	^22^
Cervical cancer	35 tumour tissues with and without PLNM+ TCGA data	Downregulated (with PLNM vs. without PLNM)	_	Shorter OS	_	Lymph node metastasis	^32^
Colorectal cancer (CRC)	48 PTAs +TCGA data	Downregulated	_	_	_	_	^15^
	116 PTAs (40 pairs selected for expression analysis)	Upregulated	_	Shorter DFS and OS	MIR100HG expression, AJCC stage, TNM stage, tumour differentiation (independent prognostic factors for DFS and OS)	TNM stage, lymph node metastasis, distant metastasis, AJCC stage, tumour differentiation	^23^
	14 PTAs + their matched lymph node or distant metastasis samples	Upregulated (lymph node and distant metastasis samples vs. primary tumour samples)	_	_	_	_	^16^
Gastric cancer	122 tumour tissues and 40 ANCTs + TCGA data (379 cases)	Upregulated	_	Shorter DFS and OS	MIR100HG expression (prognostic factor for OS)	Stage, invasion, lymph node/ distant metastasis	^24^
	56 PTAs	Upregulated	_	Poor prognosis	MIR100HG expression (independent prognostic factor)	TNM stage	^25^
	86 PTAs	Upregulated	_	_	_	_	^17^
Hepatocellular carcinoma	50 PTAs	Upregulated	_	_	_	TNM tumour stage, Edmondson‐Steiner grade	^18^
Laryngeal Squamous Cell Carcinoma	70 PTAs	Upregulated	_	_	_	AJCC stage	^19^
Oral cavity cancer	648 cases	_	rs1816158 (C allele (minor allele) carriers (CC, CT) had poor OS)	_	_	_	^33^
Osteosarcoma	88 PTAs	Upregulated	_	Shorter OS	_	Large tumour size, advanced clinical stage	^20^
Papillary thyroid carcinoma (PTC)	502 PTC patients and 58 controls (from TCGA database)	Downregulated	_	_	_	_	^34^
Triple‐Negative Breast cancer (TNBC)	20 PTAs	Upregulated	_	_	_	_	^28^
	TCGA data (4 subtypes of breast cancer)	Upregulated (TNBC vs. other subtypes)	_	Shorter OS	_	_	^29^

Abbreviations: AJCC: american joint committee on cancer; ANCTs: adjacent non‐cancerous tissues; DFS: disease‐free survival; GEO: gene expression omnibus, OS: overall survival; PLNM: pelvic lymph node metastasis; PTAs: pairs of tumour tissues and ANCTs; TCGA: the cancer genome atlas; T stage: tumour stage, TNM: tumour‐node‐metastasis.

### 2.4 Non‐malignant disorders

MIR100HG has been shown to be a crucial regulator of differentiation of human neural stem cells.^10^ It is involved in the pathology of bipolar disorder. LncRNA‐mRNA co‐expression network analysis has shown that MIR100HG is a driver gene of key modules in this disorder.^35^ Moreover, it has been among m6A‐methylated lncRNAs whose down‐regulation might be involved in the development of neural tube defect.^36^

**FIGURE 3:**
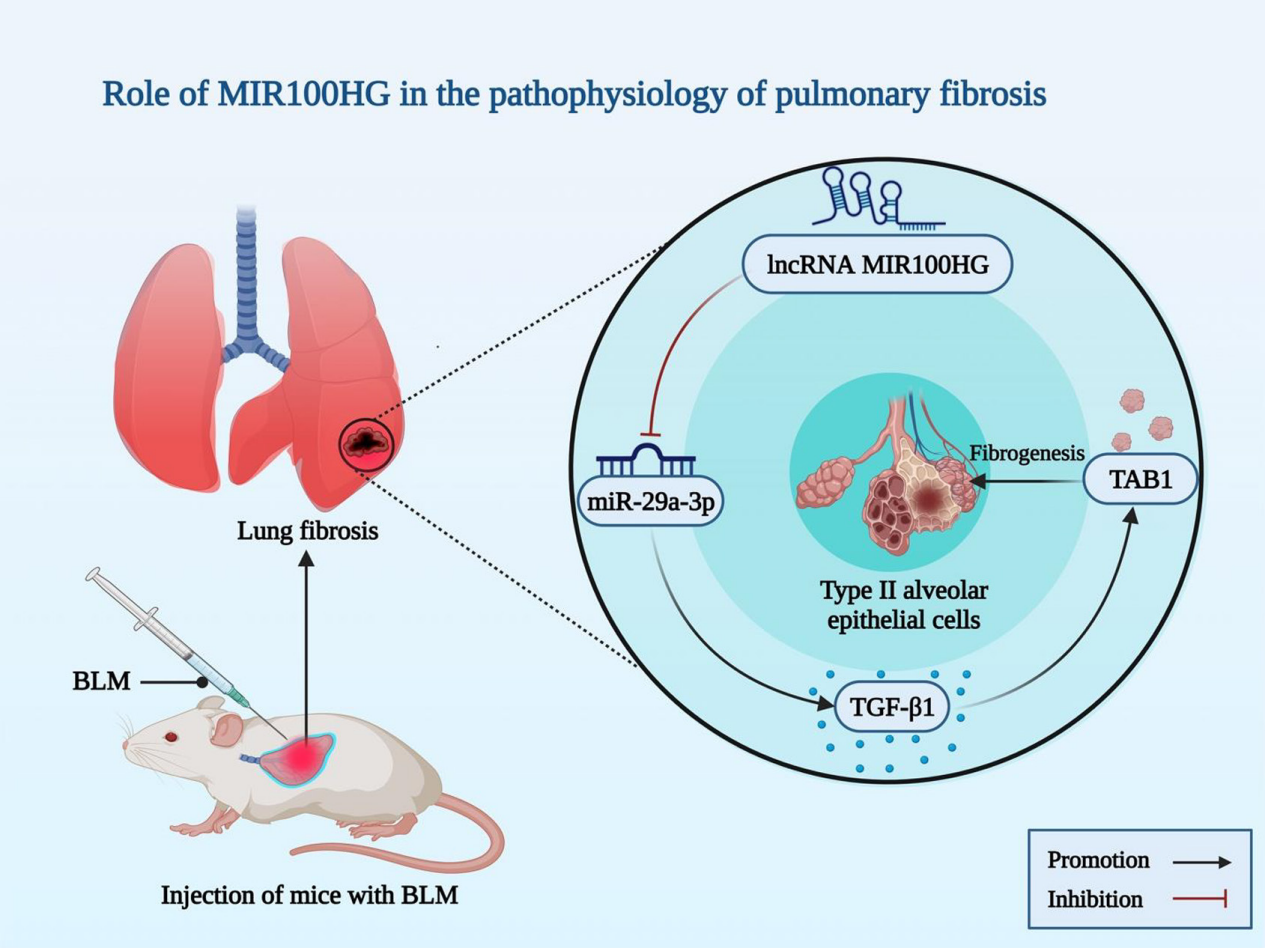
Schematic illustration representing the up‐regulation of MIR100HG in mouse type II alveolar epithelial cells triggered by TGF‐1 and bleomycin (BLM)‐induced pulmonary fibrosis, respectively. MIR100HG, which functions as a sponge for miR‐29a‐3p, has been shown to have an effect on the expression of TGF‐activated kinase 1/MAP3K7 binding protein 1 (TAB1) and cause fibrogenesis.

MIR100HG has also been found to exert a role in the pathophysiology of dilated cardiomyopathy, intervertebral disk degeneration and pulmonary fibrosis (Table 4). MIR100HG has been upregulated in lung tissues of patients with idiopathic pulmonary fibrosis (IPF). Moreover, animal and in vitro studies have indicated up‐regulation of MIR100HG in bleomycin‐induced pulmonary fibrosis and TGF‐β1‐stimulated type II alveolar epithelial cells of mice, respectively (Figure 3). MIR100HG silencing has reduced bleomycin‐induced lung fibrogenesis in animal models. Moreover, MIR100HG silencing has led to attenuation of TGF‐β1‐associated fibrotic alterations in cultured type II alveolar epithelial cells. Mecahnistically, MIR100HG sponges miR‐29a‐3p and affect expression of TGF‐β activated kinase 1/MAP3K7 binding protein 1 (TAB1). Taken together, MIR100HG has been found to be over‐expressed in bleomycin‐induced lung fibrogenesis and TGF‐β1‐stimulated MLE 12 cells. This lncRNA can affect TGF‐β1‐associated fibrotic alterations in type II alveolar epithelial cells and, therefore, can be a target for treatment of pulmonary fibrosis.^37^

**TABLE 4:** Summary of studies that reported abnormal level of MIR100HG expression in non‐cancerous diseases (GEO: gene expression omnibus).

Disease type	samples	Expression (patient group vs. controls)	Functional studies	Reference
Dilated cardiomyopathy (DCM)	12 patients and 9 controls (heart tissues)	Upregulated	‐	^38^
Intervertebral disk degeneration (IDD)	11 blood samples of patients and 7 control blood samples	Upregulated	–	^39^
Pulmonary fibrosis	40 IPF lung tissues and 8 controls (from GEO database)	Upregulated	∆MIR100HG: ↓stimulating effect of TGF‐β1 on DNA synthesis and migration, ↓TGF‐β1‐induced changes (TGF‐β1‐associated alterations: ↑ α‐SMA, collagen I, vimentin and ↓E‐cadherin)	^37^

The role MIR100HG in the pathogenesis of dilated cardiomyopathy has been indicated by a microarray study of lncRNA profiles. MIR100HG has been among eight hub lncRNAs in this module. Being located in the cytoplasm, MIR100HG has been predicted to act as a competing endogenous RNA.^38^ MIR100HG has also been among important lncRNAs that are linked with oxidative stress in the contetx of intervertebral disc degeneration.^39^

## 3 DISCUSSION

MIR100HG is a miRNA host gene whose roles in the development of diverse disorders, principally cancers, are being clarified.

The regulatory functions of MIR100HG in human disorders are exerted through modulation of genes expression at pre‐transcriptional and post‐transcriptional levels. The former is mainly exerted through acting as a promoter of RNA binding proteins and as a constituent of nucleic acid‐protein complexes. Examples of the latter function are its role as either miRNA sponges or miRNA precursors.^7^ Most notably, the effects of MIR100HG on autophagy or apoptosis are mediated through modulation of TGF‐β, Wnt, Hippo and ERK/MAPK signalling pathways mainly in an independent manner from miRNAs of same origin (mir‐125b‐1, 100, and let‐7a‐2).

Apart from a single study in bladder cancer, which has reported down‐regulation of MIR100HG in cancerous cells compared with their normal counterparts,^22^ most in vitro studies have indicated an oncogenic role for MIR100HG. Studies in clinical samples also supportive of an oncogenic role for this lncRNA, except for independent studies in bladder,^22^ cervical,^32^ colorectal,^15^ and thyroid^34^ cancers. These inconsistencies might be due to different functions of alternatively spliced transcripts from this locus. However, none of the conducted studies has mentioned the name of assessed transcripts.

MIR100HG is also involved in the induction of chemoresistance in cancer cells. Accordingly, over‐expression of this lncRNAs as well as miR‐100 and miR‐125b has been detected in cetuximab‐resistant colorectal cancer and head and neck squamous cell carcinoma cells as well as tumour samples from colorectal cancer patients that had resistance to cetuximab.^40^

Expression studies of lncRNAs in clinical samples has some difficulties including those related with isolation and analysis of lncRNAs, particularly efficiency of RNA isolation protocols, appropriate storage conditions, endogenous normalization and accuracy of RNA analysis techniques. Therefore, the inconsistencies between the results of mentioned studies in clinical samples might be at least partly explained by these technical points.

MIR100HG is functionally related with a number of signalling pathways such as TGF‐β, Wnt, Hippo and ERK/MAPK signalling pathways. Moreover, it can exert some of its roles in the carcinogenesis through sequestering miRNAs. miR‐142‐5p/CALD1, miR‐146b‐5p/CBX6, miR‐5590‐3p/OTX1 and miR‐29a‐3p/TAB1 are examples of miRNA/mRNA axes that are modulated by MIR100HG. Additional miRNAs that are sponged by MIR100HG should be identified through high throughput sequencing and functional studies. The prognostic role of MIR100HG has been verified in various cancers showing strong associations between dysregulation of this lncRNA and indicators of poor clinical outcome such as regional and distant metastasis and low level of cell differentiation.

The bulk of studies presented above indicates that essential roles of MIR100HG in the pathogenesis of human disorders are mainly exerted in an independent manner from the encoded miRNAs by this gene.

In spite of the presence of several studies on the role of MIR100HG in the carcinogenesis, its role in non‐cancerous disorders has been less studied. Therefore, upcoming studies should focus on this feature.

### 3.1 Concluding remarks

MIR100HG is an example of lncRNAs with dual functions in tumorigenesis. However, the majority of studies have designated an oncogenic role for MIR100HG. These roles are most probably independent from the functions of miRNAs that are transcribed from this locus.

## AUTHOR CONTRIBUTIONS

**Soudeh Ghafouri‐Fard:** Writing – original draft (equal); writing – review and editing (equal). **Atefeh Harsij:** Data curation (equal); formal analysis (equal); writing – original draft (equal); writing – review and editing (equal). **Hossein Farahzadi:** Resources (equal); validation (equal). **Bashdar Mahmud Hussen:** Data curation (equal); formal analysis (equal). **Mohammad Taheri:** Validation (equal); visualization (equal); writing – original draft (equal). **Majid Mokhtari:** Investigation (equal); validation (equal).

## FUNDING INFORMATION

Not applicable.

## CONFLICT OF INTEREST STATEMENT

The authors declare they have no conflict of interest.

## Data Availability

Not applicable.
